# What Asylums Were, Are, and Ought to Be

**Published:** 1838-01

**Authors:** 


### Art. V.

*What Asylums were, are, and ought to be: being the Substance of five Lectures delivered before the Managers of the Montrose Royal Lunatic Asylum.* By W. A. F. Browne, Surgeon, Medical Superintendent of the Montrose Asylum, &c.—*Edinburgh*, 1837. 8vo. pp. 231.

It must, we think, be considered a fortunate circumstance for any asylum when a book of this import is published by its superintendent. The reflections in which it must have originated cannot but have reacted favorably on the mind, and have preserved it from falling into common systems of treatment,—the offspring of indolence, inhumanity, or impatience. Of all situations, that of the superintendent of lunatics calls for the most vigilant industry, the most unwearied patience, and a humanity most proof against disappointments. No one who is practically acquainted with the character of lunatics, with their cunning, their obstinacy, and their perverseness, can be surprised to find how many keepers of lunatic-houses at length desist from curative efforts, medicinal or moral, and become content to keep their patients well fed and out of the way of accidents. But as the undoubted consequence of this quiescent method is, that many lunatics are deprived of the chances of cure, and many continue to be unjustly confined for life, the merit of a superintendent is very great whom all the troubles of an asylum cannot divert from the great duty of providing for the cure of the greatest possible number of his patients, and for the comfort of all. Regardless of their insensibility and their ingratitude, such a man is merciful to all who are placed under his control, and imitates, at humble distance, the just equanimity of a higher Power, on which he and they alike depend.

The title of Mr. Browne's first Lecture is, *What is Insanity?* and of the second, *What are the Statistics of Insanity?* The third, fourth, and fifth refer to the questions of what asylums *were*, *are*, and *ought to be.*

Unfortunately for Mr. Browne, our critical composure is disturbed at the outset, by the impossibility of digesting two diametrically opposite assertions: "I have no claim to originality," says Mr. Browne, in the second page of his preface; but, in the first page of his book, and the very first sentence, he says, "The pages which I am about to submit to you, and subsequently to the public, possess one quality which many regard as a merit, but which I am inclined to think is a misfortune. It is that of originality." Never did author commence by placing his readers in such a dilemma; from which we extricate ourselves by believing the assertion in the preface, as of later date, and made with more deliberation; to which we are furthermore inclined, as it may relieve the author from the oppressive sense of that originality which he regards as a misfortune, and the straining after which is a calamity from which he cannot always be said to escape. This admission by no means abates from his merit in bringing forward known facts in a new light, and enforcing valuable truths anew, for an important and a philanthropical purpose.

Mr. Browne appears to us to confound two things essentially different, in his chapter on the nature of Insanity. Abjuring all attempts to define insanity, which he considers to be only so many attempts "to discover one form of words expressive of the nature of a hundred different things,"

he seems to look upon such definitions as having for their great object the pointing out of the limits which make confinement or restraint necessary. It is, however, one thing to define the meaning of unsound mind, and another to point out the cases which require confinement or restraint. Mr. Browne's professed views have enabled him to pass over, unnoticed, any definitions of others; and yet, when attempting a division of the species of insanity, after quoting Arnold's and Heinroth's, he gives, as "the best," his own division: that division actually includes a definition, which he appears to consider his own, and which he repeats at least eleven times in his first chapter. We think he must have noticed something very much like this definition, and also an approach to exact rules for applying it to practical purposes, among the recent works to which he makes brief and occasional reference. We trust we can make this matter clear, with perfect fairness to Mr. Browne, by quoting his own

"ARRANGEMENT.

"I. Idiocy. Non-development of faculties.
1. Gradation. Non-development of all the powers.
2. — External senses developed.
3. — A propensity or affection developed.
4. — An intellectual power developed.

"II. Fatuity. Obliteration of faculties.
1. Partial.
2. Complete.

"III. Monomania. Derangement of one or more faculties.

*Section* I.

1. Satyriasis.
2. Homicidal and destructive.
3. Proud.
4. Vain.
5. Timid.
6. Cunning and suspicious.
7. Religious and superstitious.
8. Desponding and suicidal.
9. Imaginative.
10. Avaricious.
11. Benevolent or affectionate.

*Section* II.

12. Incapability of perceiving relations of ideas.
13. Incapability of perceiving relations of external things.
14. Incapability of perceiving qualities of external objects.

"IV. Mania. Derangement of all the faculties.
1. Mania with increased activity.
2. Mania with diminished activity." (P. 12.)

On inspecting this list, it appears evident that Section II. of Monomania applies to every case in Section I.; that it is their *definition*, and also that of all the varieties of Idiocy, Fatuity, and Mania. In all, the relations of things or of ideas are incorrectly perceived. This the author himself says over and over again, when separately describing the eleven varieties of Section I.; and he might certainly have said it all through. The conclusion virtually admitted, without notice or mention, is, that insanity consists in such a disturbance of any faculty as leads to an incapacity of perceiving the just relations of things or ideas. But how are the just relations of things or ideas perceived? By comparing one thing with another, or one idea with another. Mr. Browne will, therefore, we are sure, pardon us if we remind him that this is the definition of insanity of a previous author; the principal object of whose work was no other than to show that many who come under this definition were still not subjects for confinement; but solely those whose comparing powers were injured on such points that they could not be at liberty with safety to themselves or others, or to the property of themselves or others, or to the feelings of themselves or others. Both the definition and the rule are adopted by Mr. Browne; but he is oblivious when he regards them as new. We are not the less glad to see the definition appear in any shape, for we believe it to be true; and we are even more glad to see the rule enforced, because it alone is consistent with humanity, or even with justice.

Readers acquainted with the writings of the phrenologists will see that Section I. of the arrangement we have quoted nearly follows the phrenological arrangement of the faculties. Mr. Browne acknowledges his belief that by no other system can insanity be understood, described, or treated. We admire the boldness with which he avows his attachment to a system which is too often mentioned merely for the purposes of ridicule; and we do not hesitate to say, that we think the diversities of the mind, sound or unsound, are only explicable on the first principles of those who consider the brain as a congeries of organs. In another respect, however, we cannot at all agree with the author. Smitten, we fear, with the presumptuous philosophy of the French physiologists, he is dissatisfied with any theory of insanity which does not refer every case to lesion of the structure of the brain, and account for the degree of the malady by the degree of palpable lesion. In the majority of fatal cases, we certainly should expect something more than functional disorder; structural lesion, the result of long continued disease; but, as Mr. Browne admits that the brain may be irritated by the disorder of other organs, we think he must also admit that its mere functional disturbance may occasionally induce violent symptoms of disordered mind. When physiologists know something of the mode in which the brain acts in health, pathologists may be allowed to speak positively of its unhealthy states; but not before. When it is proved that the nervous system has no actions which the eye cannot follow, no conditions which are not appreciable by sight or touch, we may expect to find every symptom explained by hardness or softness, by redness or paleness, by hypertrophy or wasting, by extravasation or ulceration; but not before. The assertion of invariable palpable lesion of structure, wherever there is lesion of function, is no more, at present, than the jargon of a school which dreads the admission of anything that is not physical, and, on the ground of this miserable and narrow view, aspires to the dignity of a superior philosophy. The best excuse for the English students who bring it over from Paris, where it is loftily declaimed at the bedsides of the dying, amidst the acclamations of much-delighted pupils, is, that they only think it very fine because they do not comprehend it, whilst it has the advantage of discrediting the existence of many more things which they comprehend quite as little. We are far from ranking Mr. Browne in such a class: he evidently possesses far more experience and more knowledge. We can only ascribe his adoption of the French pathology to inadvertence.

Mr. Browne notices several interesting facts in his chapter on the Statistics of Insanity. The proportion of the insane to the sane, throughout Europe, is said to be 1 in 1000; in Wales, it is said to be 1 in 800; in Scotland, 1 in 574; but in America, 1 in 262. Of 472 cases given by Esquirol, only 13 arose from excess of study, 100 were ascribed to an excess in some propensity or other, and 90 to ill-regulated sentiments. In 1000 cases cited by Georget, 470 were ascribed to irregular morals, 106 to drunkenness, 20 to an ill-conducted education, and 25 to mental labour. One-half of the cases of insanity, Mr. Browne well observes, arise out of crimes, folly, and ignorance. If we add to this consideration that of the number of crimes which flow from ignorance, we cannot but deduce a very important conclusion from such a review; and if, as there appears much reason to believe, insanity prevails more in this age of intellectual activity and increasing comfort, its prevalence shows that mankind, in their advance towards worldly possessions and personal enjoyments, have not yet learned the highest wisdom, that greatest art of life, the management of the "restless mind." We believe Mr. Browne takes a correct view of this matter when he states that "the professions which are most intimately connected with temporal and selfish interests, and the dispositions which are vicious or lead to vice, are precisely those upon which the infliction falls most heavily." (P. 56.) As regards mental occupation, we do not think he is justified in attributing, as we understand him to do, as disturbing an influence to the exact sciences, so called, as to pursuits which excite the imagination. Led away, we cannot but think, by the desire of seeming very original before the trustees of the Montrose Asylum, he accuses Dr. Conolly of having "made the startling assertion that, among the educated classes of patients admitted into Bicêtre, no instances of insane geometricians, physicians, naturalists, or chemists are to be found, while priests, poets, painters, and musicians occur in great numbers." This he considers to be quite erroneous, and opposed to Esquirol's observations in his private asylum, appropriated to the wealthier and educated classes; whereas the Bicêtre is the asylum of the destitute. In M. Esquirol's asylum, it is observed, there are neither priests nor poets, but two engineers, four physicians, four chemists, &c. &c. But Mr. Browne should rather appeal to the registers of the Bicêtre themselves, for priests and poets are not unfrequently found to be poor. He has also somewhat perverted Dr. Conolly's meaning, which is best shown by his own words in the passage which we take to be the one referred to in Mr. Browne's work.

"The registers of the Bicêtre, for a series of years, show that even when madness affects those who belong to the educated classes, it is chiefly seen in those whose education has been imperfect or irregular, and very rarely indeed in those whose minds have been fully, equally, and systematically exercised. Priests, artists, painters, sculptors, poets, and musicians, whose professions so often appear marked in that register, are often persons of very limited or exclusive education ; their faculties have been unequally exercised; they have commonly given themselves up too much to imagination, and have neglected comparison, and have not habitually exercised the judgment. Even of this class, it is to be remembered that it is commonly those of the lowest order of the class, in point of talent, who become thus affected; whilst, of naturalists, physicians, chemists, geometricians, it is said not one instance occurs in these registers." (*Indications, &c.,* p. 92.)

Mr. Browne says this is accounted for by the absence of naturalists, physicians, chemists, and geometricians from the Bicêtre, in consequence of their being in comfortable circumstances; but, in the next page, he himself asserts that poets and priests are more frequently attacked by insanity than either physicians or naturalists, which is nearly all that was asserted in the assertion which he considers so startling; and he ascribes this also to the excitement of the imagination, which was all that was contended for. (P. 58.) He even quotes a table of the professions of 500 patients at Charenton, among whom are only six physicians and two chemists. There are, to be sure, no poets, and only six priests.

In another respect we think Mr. Browne incorrect: he seems to consider the agricultural poor nearly exempt from insanity. "Poverty," he says, "enjoins a compulsory temperance; it shuts out the longings of ambition; it acquaints with the realities of life, and excludes the effects of sentimentalism," &c. &c.; all which is undeniable: but when he goes on to say, that "the agricultural population, which presents poverty in its most attractive forms, and enjoys its best privileges, is to a great degree exempt from insanity," we think he is altogether mistaken. Our own opportunities of acquaintance with poor agricultural labourers, so far from having impressed us with the idea of their poverty exhibiting itself in attractive forms, leads us to consider a large proportion of them as among the most abject and the most wronged of mankind. Toiling all the day, they do not earn sufficient to enjoy decent food, clothing, or bed; and, if their children were not gratuitously clothed and instructed, many of them would be but naked savages. Of the diseases treated in country dispensaries, we have found about two-thirds to be some of the forms of dyspepsia, the product, we believe, of bad food, misery, and care, and exposure to weather. Among such people, we should expect no immunity from insanity, and we find none. Not a village without its mad people. Not a common asylum throughout the country without its complement of wretched labourers and labourers' wives, scarcely in the rank of civilized creatures. Such impressions, we know, are not to be taken in evidence as calculations; but even calculations are not wanting. Dr. Prichard, in his valuable Treatise on Insanity, makes the following observations:

"Some curious facts develope themselves in regard to the comparative frequency of madness and idiotism in different ranks of the community. Of the 14,000 insane calculated to exist in England, or of the 12,547 ascertained, not fewer than 11,000 are paupers, maintained principally at the expense of parishes. A most remarkable difference is found in the proportional number of lunatics in agricultural and in manufacturing districts. Previous to enquiry, we should conjecture that the causes of insanity would have more influence, and the disease be more prevalent, in a manufacturing, than in an agricultural population; but the contrary is the fact. Thus, in twelve counties in England, of which the inhabitants are chiefly employed in agriculture, the entire population being 2,012,979, the insane amount to 2526, giving about 1 lunatic to 820; while in twelve counties where the majority of the inhabitants are otherwise employed, including Cornwall, where a great number are miners, the entire population being 4,493,194, the insane amount to 3910, or nearly as 1 to 1200. In Scotland and in most of the Welsh counties the population is chiefly agricultural, and this may, perhaps, account for the greater proportion of lunatics in the population of those parts of the island." (*Dr. Prichard's Treatise*, p. 334.)

Dr. Prichard thinks it probable that the labouring of women in the field during pregnancy, may, as suggested by Sir A. Halliday, be one cause of this disproportion; and that hard labour and low diet may have some influence. It is to be observed that Mr. Browne quotes Halliday, Esquirol, and Duncan, in support of his very opposite opinions concerning this question. The chapter on Statistics contains several curious particulars, worthy of perusal, and concludes, not very appropriately, but by a defective arrangement very prevalent in these lectures, with remarks on the employment of lunatics as a means of cure. The remarks themselves are excellent.

In Lecture III. we reach the real commencement of the book, according to its title; the heading of this chapter being *What Asylums were.* And truly a very instructive chapter it is. It should be carefully read by all visiting magistrates, especially, and visiting physicians appointed at quarter-sessions; whose duties are generally ill performed; partly because they do not know their own powers or the exact nature of their office, and partly from local influences. We well know that asylums are not now what they were: but they were what they were for want of superintendence; and to whatever points superintendence cannot reach, human nature being the same, it is always to be feared corruption will yet extend, and negligence. There are mercenary establishments in every county. Nothing is so easy as entrance there; nothing so tardy and difficult as deliverance.

The shocking details given by Mr. Browne are quoted from authentic documents, from the Reports of Parliamentary Committees chiefly; and, bad as they are, we think that worse might be adduced. Their force is somewhat weakened by a kind of challenging and declamatory style, arising out of a redundancy of Scottish energy, and which characterizes Mr. Browne's book; but the facts are sufficiently horrible. The author sums them up as follows:

"Let us pass a few minutes in an asylum as formerly regulated, and from the impression made by so brief a visit, let us judge of the effects which years, or a life-time spent amid such scenes, was calculated to produce. The building was gloomy, placed in some low confined situation, without windows to the front, every chink barred and grated—a perfect goal. As you enter, the creak of bolts, and the clank of chains are scarcely distinguishable amid the wild chorus of shrieks and sobs which issue from every apartment. The passages are narrow, dark, damp, exhale a noxious effluvia, and are provided with a door at every two or three yards. Your conductor has the head and visage of a Charib; carries, fit accompaniment, a whip and a bunch of keys, and speaks in harsh monosyllables. The first common room you examine, measuring twelve feet long, by seven wide, with a window which does not open, is perhaps for females. Ten of them, with no other covering than a rag round the waist, are chained to the wall, loathsome and hideous; but, when addressed, evidently retaining some of the intelligence, and much of the feeling which in other days ennobled their nature. In shame or sorrow, one of them perhaps utters a cry; a blow which brings the blood from the temple, the tear from the eye, an additional chain, a gag, an indecent or contemptuous expression, produces silence. And if you ask where these creatures sleep, you are led to a kennel eight feet square, with an unglazed air-hole eight inches in diameter; in this, you are told five women sleep. The floor is covered, the walls bedaubed with filth and excrement; no bedding but wet decayed straw is allowed, and the stench is so insupportable, that you turn away and hasten from the scene. Each of the sombre colours of this picture is a fact. And those facts are but a fraction of the evils which have been brought home to asylums as they were." (P. 132.)

In proceeding to speak of *What Asylums are*, which forms the subject of the Fourth lecture, there are some pages of a verbose declamation which should surely have been retrenched before publication. "The cry of the lunatic uttered in the exuberance of his own self-inflicted anguish," and similar passages of oratory, whatever may have been thought of them at Montrose, go far to throw ridicule over Mr. Browne's best intentions. His style is remarkably round-a-bout. Meaning to express that the evils of the past and present condition of the insane may have been exaggerated, he says, "exaggerations have unquestionably crept in, and become amalgamated with the rigid bare truth of many of the statements which have been advanced, both as to the past and present condition of the insane. But, has the apocryphal no parallel in the accredited history? Ignorance—ignorance alike of all that could and of all that did befall within an asylum lent its aid." Again, intending to say that a progressive improvement is taking place in asylums, he says, "universally a decided change is contemplated and desired, and the din of preparation is heard wherever isolation is attempted."

In these, as in many other instances, Mr. Browne's style becomes ineffective by aiming at the forcible; and his lectures acquire a kind of colouring, as if intended to astonish more than to teach. We should not mention this fault, if whole pages were not sometimes disfigured by it, and often much to the obscuring of their sense and meaning. Mr. Browne quotes from our first Number the account of Pinel's glorious emancipation of the enchained lunatics at the Bicêtre in 1792; from which event we believe he correctly dates the beginning of the improved treatment of insane persons which characterizes modern times. This simple and impressive fact he, as usual, somewhat overloads with eloquence. "From darkness," (he exclaims, meaning, we conjecture, medical men and legislators,) "they passed into light,—from savage ferocity into Christian benevolence. These terms are energetic;" &c. The truth is, these terms are not energetic, and that is Mr. Browne's continual mistake.

But of these trifling blemishes we entirely lose sight when we read Mr. Browne's fearless exposure of the still existing evils of asylums.

"There is no classification, no employment, no exercise. If you pass through an establishment, all may be tranquil, orderly, and humane; but the inmates are lethargically slumbering on chairs, or endeavouring to devise occupation by tormenting their fellows, or circumventing their keeper. Men have been proved to have remained seated in the same spot, during the day, for a dozen years, without an attempt being made to rouse their muscular or mental energies. This species of negligence is often presented in a more hideous form. Patients who at first are perfectly able to walk, are allowed to remain in bed; their limbs waste, contract, are partially anchylosed, and they ultimately become unable to rise." (P. 144.)

We know this to be a correct picture, not of the best managed private asylums, but of the worst managed asylums of England in the present day. We know, too, that into such asylums nervous patients may be sent any day, with a regular certificate, or without one; without one properly signed, or with one signed by medical men called in merely to sign it. When once so imprisoned, all appeals for a time are vain. If the visiting physician is written to, he declines to act; if the magistrates, they make a pompous, formal, useless enquiry; if the Lord Chancellor, he has no control in the case; if the metropolitan commissioners, they order an investigation, which the magistrates can render as insignificant and useless as their first enquiry.

"Where peculation no longer exists, and where the lunatic is comfortably lodged and sufficiently clothed, there is still great inattention to the mode in which the building where he sleeps is heated. In winter he is compelled to pass, as if in imitation of a Russian bath, from the temperature of a crowded and probably over-heated common-hall, to that of a damp cell which has been cooled down by the indispensable process of ventilation to the freezing point." (P. 144.)

"In 1820, the Commissioners found a patient in a private institution, alone in an out-house, without a fire,—the visit was paid in winter—the windows were broken, probably by his own act. He was without shoes, but was in other respects sufficiently clothed. After much prevarication and deception, it was proved that this patient did not sleep in the apartment said to be his, but in a miserable room up a private stair, concealed by a door, which was discovered with considerable difficulty. It was a single room, small and offensive, containing only a wet and dirty piece of sacking filled with straw, with one rug and a blanket. For this treatment the patient paid 50*l.* per annum." (P. 145.)*

"Coercion is employed unnecessarily. Either from the savage philosophy of terrifying into obedience, and, it is to be presumed, into the possession of reason, or from the despicable economy of employing a small corps of keepers; chains, muffs, manacles, are in many places the substitutes for mildness and prudence, or suitable attendance. Not only the violent and the destructive but the perverse, even the restless and noisy maniac must be secured." (P. 146.)

"It appears that in one instance three keepers were expected to guide, govern, and soothe 250 patients. In another asylum, 164 patients were intrusted to two keepers. In a third, each servant was appointed to take charge of fifty patients. The proportion usually is one keeper for thirty lunatics. This means that one man or woman is to attend to all the wants and wishes, regulate the employments and amusements, counsel, tranquillize, walk and converse with, feed, clothe, and put to bed thirty persons, every one of whom displays a different form of insanity, is furious or fatuous, malicious or melancholy." (P. 147.)

We need not quote any of Mr. Browne's remarks on the inefficiency of the generality of attendants to do all that the peculiar state of many lunatics requires, in all stages of excitement, depression, and convalescence; nor on the imperfect classification of patients. These subjects have often, and powerfully, been brought before the attention of the public. As nothing is more to be expected than that the proprietors of lunatic asylums will accuse Mr. Browne of making exaggerated statements, we regret that he has quoted (p. 161) the erroneous opinion of some physician whom he does not name, that the indecent language used by lunatic females even of the higher classes is learned in asylums. There can be no doubt that it was learned long before, in youth, in childhood, at school, or from servants; and remaining unexpressed in a state of sanity, breaks forth in the violence of morbid excitement. It is, at the same time, a shocking revelation of the female mind, polluted early, and only glossed over by the refinement of artificial life.

Several useful observations are scattered through this lecture, as indeed through all; but whilst some refer to asylums as they are, some refer to them as they were, and some to what they should be. Instances of cruelty under the old system, and observations on the indiscriminate diet

* Report, Pauper Lunatics in Middlesex, p. 158, 1827.

in existing asylums, are mingled together without arrangement, and the lecture concludes with a proposition to make all asylums Public Asylums, which Mr. Browne speaks of as original, although he can scarcely be ignorant of its having been proposed seven years ago by another writer.

The concluding chapter of the work is entitled *What Asylums ought to be;* and is introduced by one of Mr. Browne's strangest sentences. "A perfect asylum may appear to be a Utopia; 'a sight to dream of, not to see.' It would be miserable policy to gratify the ambition of the heart so far, or to pall the keen appetite for doing good by admitting that any attempt had succeeded in placing such retreats in complete accordance with the necessities of the diseased mind." After two readings, one may understand this, and then learn no more than the whole of the preceding lecture was intended to teach us. Mr. Browne then draws, with good discrimination, the moral and intellectual character of a physician fitted to take charge of the insane. He points out the requisite qualities of a site for an asylum, often, he justly says, too much neglected; and insists strongly and properly on more attention being paid than is common to the size and ventilation of the sleeping apartments, and to night-classification, an ample supply of baths, &c. His observations on warming asylums; on the clothing of the patients; on planting and diversifying the airing grounds; on the institution of gardens, a farm, and workshops; are well deserving of attention. He urges the propriety of remunerating the lunatic for his labour, or of letting the remuneration accumulate for him on his release; a suggestion of which the value will be appreciated by all who remember M. Esquirol's observation on the frequent relapses caused in patients by finding, on going out of the asylum, that their affairs have gone to ruin. We would also particularly recommend the observations on the mode of classifying lunatics so that the peculiarities of one may be salutary antagonists to the peculiarities of another; although we can gather that the author's opinion is, what our own assuredly has always been, that, as much as possible, every convalescent lunatic, if not every lunatic, should be kept from lunatic society. His remarks on permitting lunatics to partake, as far as their condition permits, of the consolation of attending the public services of religion, are marked by good sense and good feeling; and we entirely concur in his judicious observations on the artifices of cure which have occasionally been successful with hypochondriacal patients. We should say, with Esquirol, that such artifices should only be a last resource. With respect to secret methods of cure, like all other systematic frauds, they can only be spoken of with abhorrence. Alluding to an account given in the Medical Gazette of a ball at the Salpêtrière in May 1835, Mr. Browne says that something of the kind has taken place every week, for some time past, in the establishment under his care.

The asylum in the neighbourhood of Paris under the care of Drs. Falret and Voisin, that of M. Esquirol at Ivry, and the Retreat at Hartford, U. S. are spoken of, we believe correctly, as combining nearly every possible advantage of such institutions. In the report of the latter, it is stated that of twenty-three recent cases admitted in one year, twenty-one recovered. That at Sonnenstein in Saxony is also referred to, of which an account has been given in Dr. Burrows's Commentaries, and in Mr. Lee's work on the Medical Schools of the Continent. An account of the asylums of Naples is taken from Willis's Pencillings by the Way. Mr. Browne concludes his last lecture, and we shall conclude our notice of his book, by the following idea of an asylum as it ought to be, which we merely abbreviate by the omission of a few lines.

"Conceive a spacious building resembling the palace of a peer, airy, and elevated, and elegant, surrounded by extensive and swelling grounds and gardens. The interior is fitted up with galleries, and workshops, and music-rooms. The sun and the air are allowed to enter at every window; the view of the shrubberies and fields, and groups of labourers, is unobstructed by shutters or bars; all is clean, quiet, and attractive. The inmates all seem to be actuated by the common impulse of enjoyment, all are busy, and delighted by being so. The house and all around appears a hive of industry. When you pass the lodge, it is as if you had entered the precincts of some vast emporium of manufacture; labour is divided, so that it may be easy and well performed, and so apportioned that it may suit the tastes and powers of each labourer. You meet the gardener, the common agriculturist, the mower, the weeder, all intent on their several occupations, and loud in their merriment. The flowers are tended, and trained, and watered by one; the humbler task of preparing the vegetables for the table is committed to another. Some of the inhabitants act as domestic servants, some as artisans, some rise to the rank of overseers. The bakehouse, the laundry, the kitchen, are all well supplied with indefatigable workers. In one part of the edifice are companies of straw-plaiters, basket-makers, knitters, spinners, among the women; in another, weavers, tailors, saddlers, and shoemakers, among the men. For those who are ignorant of these gentle crafts, but are strong and steady, there are loads to carry, water to draw, wood to cut, and for those who are both ignorant and weakly, there is oakum to tease and yarn to wind. There is in this community no compulsion, no chains, no whips, no corporal chastisement, simply because these are proved to be less effectual means of carrying any point than persuasion, emulation, and the desire of obtaining gratification. But there are gradations of employment. You may visit rooms where there are ladies reading, or at the harp or piano, or flowering muslin, or engaged in some of those thousand ornamental productions in which female taste and ingenuity are displayed. You will encounter them going to church or to market, or returning from walking, riding, and driving in the country. You will see them ministering at the bedside of some sick companion. Another wing contains those gentlemen who can engage in intellectual pursuits, or in the amusements and accomplishments of the station to which they belong. The billiard room will, in all probability, present an animated scene. Adjoining apartments are used as news-rooms; the politicians will be there. You will pass those who are fond of reading, drawing, music, scattered through handsome suits of rooms, furnished chastely, but beautifully, and looking down upon such fair and fertile scenes as harmonize with the tranquillity which reigns within, and tend to conjure up images of beauty and serenity in the mind which are akin to happiness. But these persons have pursuits, their time is not wholly occupied in the agreeable trifling of conning a debate, or gaining so many points. One acts as an amanuensis, another is engaged in landscape painting, a third devolves to himself a course of historical reading, and submits to examination on the subject of his studies; a fourth seeks consolation from binding the books which he does not read. In short, all are so busy as to overlook, or all are so contented as to forget their misery.

"Such is a faithful picture of what may be seen in many institutions, and of what might be seen in all, were asylums conducted as they ought to be." (P. 229.)

This description, be it remembered, is not that of a theorist, or of an enthusiast, but of a practical man, long accustomed to the management of lunatics, and at the head of an establishment which we doubt not possesses, under his humane and skilful care, all the advantages which he has described, and one to which he has not alluded, that of having so kind and intelligent a superintendent.

